# Target Trial Emulation: Improving the Quality of Observational Studies in Inflammatory Bowel Disease Using the Principles of Randomized Trials

**DOI:** 10.1093/ibd/izae131

**Published:** 2024-06-11

**Authors:** Sailish Honap, Silvio Danese, Laurent Peyrin-Biroulet

**Affiliations:** INFINY Institute, Nancy University Hospital, F-54500 Vandœuvre-lès-Nancy, France; School of Immunology and Microbial Sciences, King’s College London, London, UK; Department of Gastroenterology, St George’s University Hospitals NHS Foundation Trust, London, UK; Department of Gastroenterology and Endoscopy, IRCCS San Raffaele Hospital, Vita-Salute San Raffaele University, 20132 Milan, Italy; INFINY Institute, Nancy University Hospital, F-54500 Vandœuvre-lès-Nancy, France; Department of Gastroenterology, Nancy University Hospital, F-54500 Vandœuvre-lès-Nancy, France; INSERM, NGERE, University of Lorraine, F-54000 Nancy, France; FHU-CURE, Nancy University Hospital, F-54500 Vandœuvre-lès-Nancy, France; Groupe Hospitalier privé Ambroise Paré - Hartmann, Paris IBD Center, 92200 Neuilly sur Seine, France; Division of Gastroenterology and Hepatology, McGill University Health Centre, Montreal, Quebec, Canada

**Keywords:** target trial, randomized controlled trial, inflammatory bowel disease, causal inference

## Abstract

The past decade has seen a substantial increase in the number of randomized controlled trials (RCTs) conducted in inflammatory bowel disease (IBD). Randomized controlled trials are the gold standard method for generating robust evidence of drug safety and efficacy but are expensive, time-consuming, and may have ethical implications. Observational studies in IBD are often used to fill the gaps in evidence but are typically hindered by significant bias. There are several approaches for making statistical inferences from observational data with some that focus on study design and others on statistical techniques. Target trial emulation is an emerging methodological process that aims to bridge this gap and improve the quality of observational studies by applying the principles of an ideal, or “target,” randomized trial to routinely collected clinical data. There has been a rapid expansion of observational studies that have emulated trials over the past 5 years in other medical fields, but this has yet to be adopted in gastroenterology and IBD. The wealth of nonrandomized clinical data available through electronic health records, patient registries, and administrative health databases afford innumerable hypothesis-generating opportunities for IBD research. This review outlines the principles of target trial emulation, discusses the merits to IBD observational studies in reducing the most common biases and improving confidence in causality, and details the caveats of using this approach.

**Key Messages**

- Observational studies are often used in inflammatory bowel research to answer questions where conducting a randomized trial is unfeasible, although they are usually laden with bias.
- This review discusses a novel concept of using existing observational data to mimic the principles of an ideal or “target” randomized trial that would ideally address the hypothesis in question.
- By reducing bias, this emerging approach has the potential to improve the quality of observational studies to test the effect of IBD interventions where RCTs are impractical.

## Introduction

The accepted standard of generating evidence to determine causal inference is to conduct a blinded randomized controlled trial (RCT). Randomized controlled trials remain at the top of the epidemiological hierarchy of study designs in clinical research due to fundamental principles that help reduce bias and maximise internal validity. These include, for example, randomization to equally distribute known and unknown confounding factors between intervention groups, synchronizing treatments assigned with the start of follow-up (time zero), concealing allocation, conducting intention-to-treat analyses, and blinding when appropriate.^1^ The first RCT conducted in gastroenterology in the 1950s established the efficacy of intravenous corticosteroids for acute severe ulcerative colitis.^2^ Since then, well-designed RCTs have been the gold standard method for generating evidence on the efficacy and safety of investigational drugs and to enable regulatory approval.

Clinical researchers in IBD should strive for methodological rigor to produce evidence of the highest grade. However, it is unfeasible to conduct an RCT to investigate all hypotheses as trials are a costly, time-consuming, and a resource-intensive endeavour, which may also have ethical implications. Observational studies in IBD are often used to bridge gaps in evidence, but there are challenges when using these data to make causal inferences due to significant biases that can result from the absence of experimental principles, particularly confounding.^3,4^ There are several approaches for making statistical inferences from observational data, with some that focus on study design and others on statistical techniques.^5^

Target trial emulation is a novel method that can improve the strength of observational studies by emulating key principles of randomized trials to provide a better measure of causal inference than mere association.^6^ The process involves detailing the protocol of an ideal clinical trial that would address the hypothesis in question and then applying these steps to the available observational data, such as those collected from routine clinical practice. The past decade has seen a significant expansion of intervention trials in IBD, mirrored by an increase in observational studies. The wealth of nonrandomized data also available through registries, claims databases, and electronic health records of IBD patients also provide ample opportunities to utilize this novel approach for observational studies and improve the validity of conclusions drawn.

Target trial emulation has been successfully used in other medical fields over the past 5 years to improve study quality, notably in cardiology, oncology, and infectious diseases.^7^ While there are no published studies in the field of IBD, it is important for the IBD community to be aware of this emerging approach that may hold important utility. This review outlines the principles of target trial emulation, discusses the advantages it can afford to IBD observational research in reducing bias and producing effect estimates comparable to RCTs, and details the limitations of using this approach.

## Target Trial Emulation: An Overview

**Figure 1.**
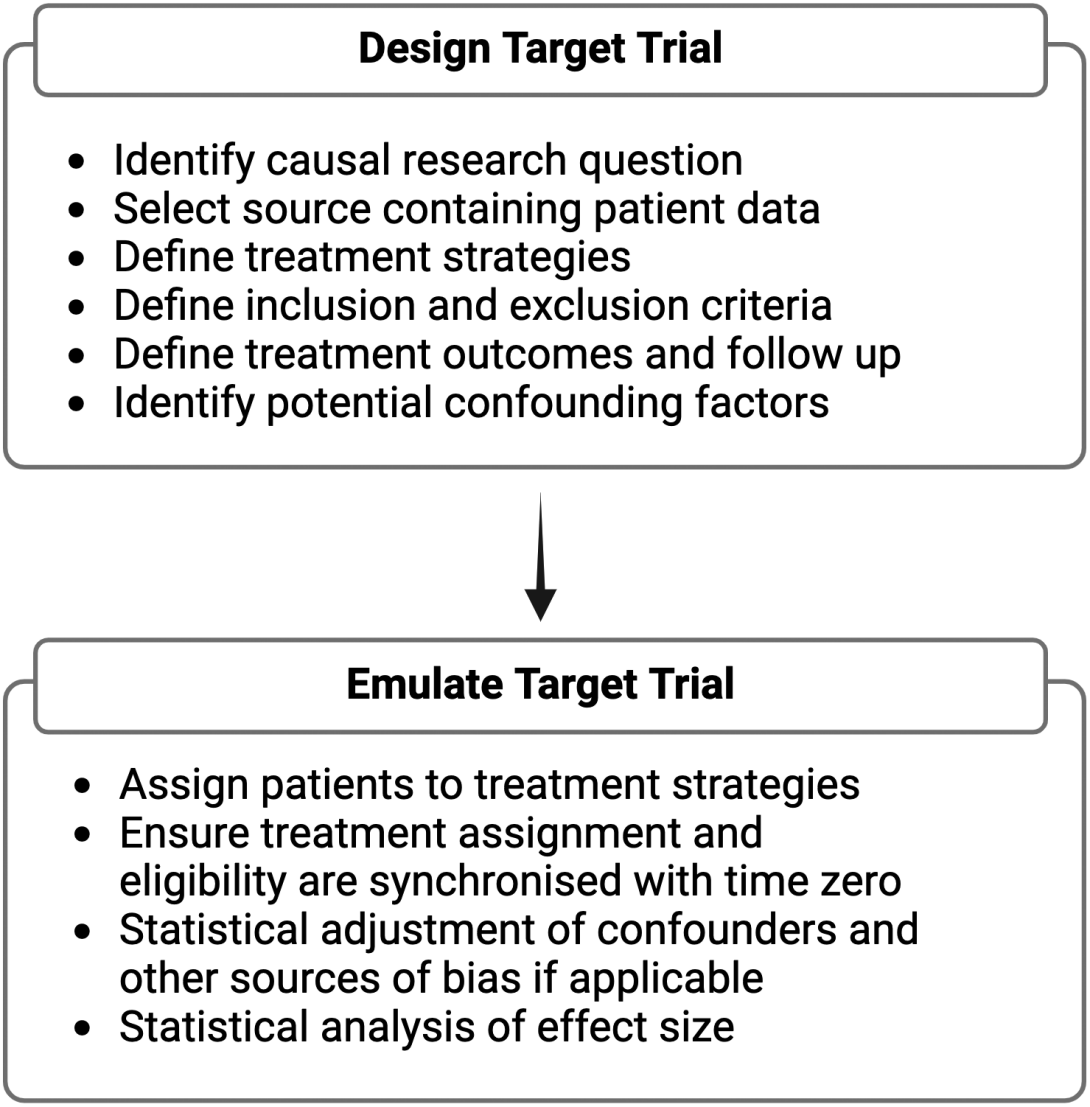
Target trial emulation workflow framework.

Although the term “target trial emulation” was described in 2016, the first study that applied this concept dates back to 2008 when researchers emulated the design and analysis of a randomized trial of postmenopausal hormone therapy and coronary heart disease using observational data.^6,8^ The framework of emulating a target trial that has since been proposed is composed of 2 key stages—protocol design and protocol implementation (Figure 1).

### Protocol Design

The initial stage of target trial emulation involves developing a protocol of an ideal randomized trial that would adequately address a clear causal question regarding an intervention. The target trial protocol can then be modified to accommodate the nature and limitations of the available clinical data to be used. The target trial can be based on an actual trial (previously conducted or currently ongoing) or a hypothetical trial, the conduct of which does not necessarily need to be feasible. Further, as this approach utilizes already collected data rather than involving human subjects directly, it is not constrained by the same ethical requirements as RCTs. Nonetheless, such studies using retrospective or prospectively maintained data should be conducted in accordance with the local legislation and institutional requirements.

The trial protocol should carefully delineate aspects of the trial that need to be emulated, including the data source to be used, eligibility criteria, intervention assignment, outcome measures, and defining the start and end of follow-up (Table 1).^9^ There should also be a statistical plan outlining how the outcome data will be analyzed and how sources of bias will be handled.

**Table 1.** Components of a target trial protocol for an observational study in inflammatory bowel disease.

Protocol Component	Target Trial	Observational Study
Data source	Not applicable—no external data source is required to deliver a randomized trial	Outline planned data sources, whether these will include structured or unstructured data, and whether natural language processing will be used to retrieve and extract data. Data may be derived from observational studies, patient registries, electronic health records, health insurance claims.
Eligibility criteria	Inclusion and exclusion criteria should be clearly defined eg, the eligibility criteria typically used in a phase III pivotal IBD trial or variations of this	Identify and enroll patients at the time eligibility criteria of target trial is met and before treatment has commenced. Data collected during follow-up cannot be used to determine eligibility.
Intervention	Patients are randomized to treatment arms during the induction and maintenance phase. Description of intervention—dose, exposure duration, regimen during course of trial, and discontinuation rules.	Assign patients to the intervention (depending on the drug prescribed), which should mirror target trial. Define what is being assessed: point treatment vs treatment regimen and induction vs maintenance.
Time zero	Time zero (treatment assignment and start of follow-up) starts at point of randomization (Figure 2). Follow up should be described—whether this is at the occurrence of endpoint, censoring, end of follow-up.	Time zero must be clearly specified. The time the eligibility criteria is met should be synchronized with treatment assignment (medication initiation). Follow up to mirror target trial.
Bias	Significant sources of bias are addressed by process of randomization.	Outline known confounders and how they will be adjusted for. Describe whether immortal bias was handled at study design stage (mentioned previously) and if not, what technique was used to adjust. Outline how selection bias was handled.
Outcome	Primary and secondary outcomes should be defined as well as how effect size was compared (mean difference odds ratio, hazard ratio etc). Blinded assessment of outcomes (eg, central endoscopy reading).	Outcome definitions should match. Ability to emulate number of outcomes dependent on data availability. Lack of blinding introduces bias.
Statistical analyses	Intention-to-treat analysis to assess causal estimation	Intention-to-treat analysis or per-protocol analysis to estimate treatment effect
Reporting guideline	Consolidated Standards of Reporting Trials (CONSORT)	Strengthening the Reporting of Observational Studies in Epidemiology (STROBE)

### Protocol Implementation

The next stage involves closely following the protocol to conduct an observational study that emulates the target trial. The source of observational data, such as electronic healthcare records, should be searched to identify patients that match the eligibility criteria. Patients are then assigned to a treatment (based on the treatment they were retrospectively prescribed), followed from the point of assignment (time zero) to the outcome or end of follow-up, and completed with analyses of the causal estimand.^9^

### Reducing Bias With Target Trial Emulation

In an RCT, treatment groups are comparable such that the outcome effects can be attributed to the intervention rather than other factors. For observational studies, a variety of statistical methods need to be employed to reduce the 3 main sources of bias—these include confounding bias, immortal time bias, and selection bias.^10^ A detailed discussion regarding statistical methods is outside the scope of this review but is covered comprehensively elsewhere.^11^

To mimic the process of randomization, all potential confounding factors need adjusting, while accepting that it is not possible to address unknown confounders. Importantly, this is only possible if the observational data source has sufficient details of these factors. Methods to account for confounders are to include them in the statistical model, to use propensity score methods (matching, stratification, adjustment), or to use g-methods (inverse probability weighting and parametric g-formula).^11^

**Figure 2.**
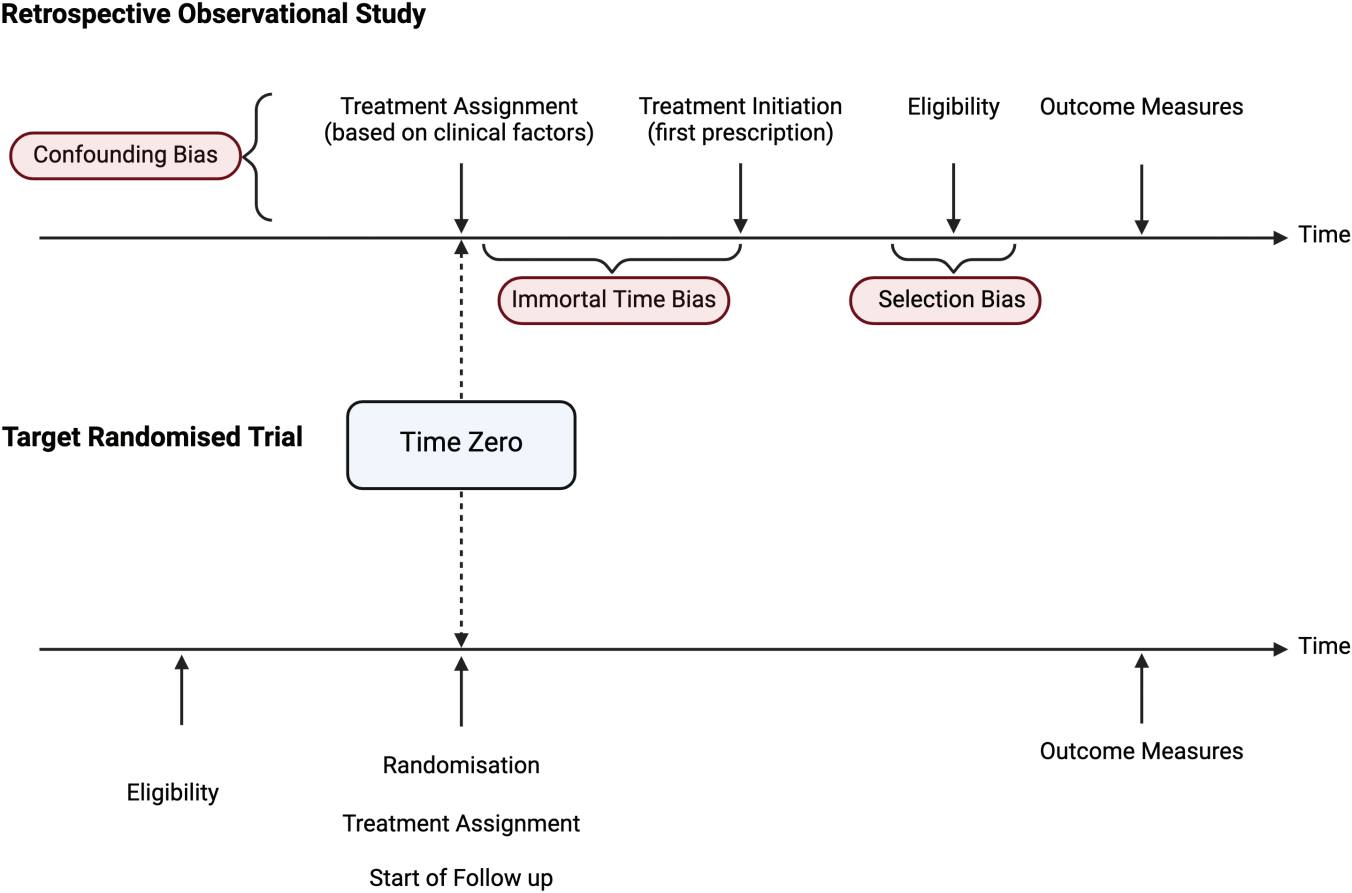
Minimizing bias with target trial emulation and the concept of time zero. A, Retrospective observational study (an example of bias introduction). Patients are prescribed treatments based on a number of demographic and clinical characteristics (for example, age, comorbidities, disease type, disease severity), which leads to an unequal distribution across treatment groups and confounding bias. Therefore, confounding factors should be identified and statistically adjusted to reduce bias. Delays in initiating treatment (eg, prescription delays) leads to a period where the study outcome cannot occur and is referred to as “immortal time.” By misaligning time zero, the specification of the eligibility criteria, the treatment assignment and actual treatment initiation can result in immortal time bias. Finally, selecting patients after treatment assignment (and availability of some study outcomes) may lead to selection bias. B, “Target” randomized controlled trial. Participants are first screened based on eligibility criteria, randomly assigned to treatment, and then followed up for outcomes. Randomization ensures equal distribution of measured and unmeasured confounders. Eligibility assessment occurs prior to randomization and patient follow-up starts at the point of randomization. Time zero (baseline) refers to the date when an eligible patient initiates treatment and study follow-up begins—this aligns naturally for RCTs where time zero is the time of randomization and minimizes bias.

Immortal time bias is common in observational studies but by way of study design, is avoided in RCTs. It occurs if the start of follow-up occurs before treatment assignment, as subjects assigned to the treatment group have a period of follow-up during which they cannot experience the outcome and are considered “immortal.” Therefore, the time at which a patient is found to be eligible must coincide with the time they are assigned a treatment and followed up (time zero; Figure 2). Time zero should be defined with data available at that specific time timepoint and not guided by later treatment data. If this is not possible, there are 2 other ways to deal with this bias. The first includes cloning, where 2 exact copies of each individual are created and followed when it is not possible to assign them to a treatment at the outset. The second includes sequential target trial emulations, which is used when patients may meet the eligibility criteria of the target trial at separate time points.^10,12^

Finally, enrolling patients once outcome results become available may lead to selection bias (Figure 2), which can be avoided with a target trial emulation approach. Other sources of selection bias must also be considered; for example, there should be a description regarding how missing data or patients lost to follow-up were handled.

## Opportunities for Inflammatory Bowel Disease Research

Target trial frameworks are ideally suited to estimating the causal effect of 1 or more interventions using observational data, which affords several opportunities for IBD research. The use of observational data is gaining traction as a valuable tool in healthcare to inform decisions on the effectiveness and safety of interventions in clinical practice as well as for health care policy and health technology assessments. Although methods to reduce bias in observational studies in IBD are increasing, such as propensity score matching to adjust for covariates, it does not systematically reduce bias in the same way as target trial emulations.^13–15^ Despite the publication of the strengthening the reporting of observational studies in epidemiology (STROBE) almost 2 decades ago, most observational studies still lack a satisfactory discussion of confounding and other biases.^16–18^ Moreover, there are some components of target trial emulation that are not included in STROBE, such as the importance of defining time zero or assessing causal contrasts.

Target trial emulation can utilize existing sources of observational data, thereby reducing cost and increasing efficiencies of generating evidence. There are 2 main data sources of interest. The first includes data compiled for research purposes, for example, ongoing or previously conducted observational studies, including cohort, case-control, and cross-sectional studies.^19–21^ A wealth of data can also be derived from the increasing number of IBD patient registries and IBD biobanks, providing carefully phenotyped patient-level data with detailed longitudinal treatment effectiveness and safety outcomes.^22–26^ The second includes data from electronic medical records and health insurance claims databases, which are not specifically generated for research purposes but often used for “real-world” studies.^27^ While this can include structured as well as unstructured data, advances in natural language processing (NLP) may aid rapid data extraction from healthcare records.^28,29^ Examples of NLP in gastroenterology include a model that was able to diagnose eosinophilic esophagitis using retrospectively extracted clinical and esophagogastroduodenoscopy data, and a model that was able to identify IBD patients on thiopurines with algorithm-predicted objective remission.^28,30^ An increase in the use of this study design may help encourage better coding of routinely collected healthcare data.

Target trial emulation can improve the quality of IBD research and can reach similar conclusions as RCTs when design and measurements are closely emulated.^31^ The RCT landscape in IBD has seen significant challenges over the past decade and a declining patient enrolment.^32,33^ In part, this is driven by an increasing competition for similar pools of patients with IBD due to a rapid expansion in novel therapies at phases 2 and 3. There is, therefore, an increasing reliance on observational studies to address research questions given the difficulties recruiting an RCT to target enrollment would entail in the current climate. Indeed, the literature is rich with observational studies investigating the effectiveness of the increasing number of treatment options available and the effect of risk factors on incident IBD and disease course. However, such studies in IBD are susceptible to significant bias and spurious findings given the lack of clarity regarding disease etiopathogenesis and the complex interplay between environmental and genetic factors. In IBD, bias reporting has been shown to be inadequate, and its acknowledgment is often neglected in interpreting high-impact observational research in IBD.^4^ Trial emulation, by default of its study design, requires researchers to consider the fundamental aspects of high quality designs and sources of bias.^31^ Further, generating and reporting the protocol of emulating a trial, increases transparency, encourages constructive feedback, and has been used as a way to identify limitations of work already conducted.^34^

Target trial emulation can interrogate existing IBD databases for other key areas of unmet research needs. Firstly, it can test the effectiveness of interventions in understudied patients with IBD where there are a lack of licensed drug indications. Several IBD patient subgroups, particularly those on the opposite ends of the spectra with respect to age and disease severity, are typically excluded from randomized trials and are poorly studied. This group includes those with multitreatment refractory IBD, stricturing, fistulizing, or perianal Crohn’s disease, and those with prior intestinal surgery and permanent stomas. Study designs to robustly test the effect of established interventions where trial data are lacking and where RCTs have not been possible are needed. Second, in the area of comparative effectiveness and safety research. The rapid innovation in advanced therapies for moderate to severe IBD means that it is increasingly difficult for clinicians to position therapies in treatment algorithms. This is due to a distinct lack of head-to-head trials and a reliance on indirect evidence from network meta-analyses of RCTs and real-world studies of varying quality.^35–37^ Finally, target trial emulation has shown promise in estimating the effect of environmental exposures (eg, diet) on health outcomes.^38^ The effect of environmental exposures on IBD pathogenesis and disease course is a highly research active area, although observational studies so far are hindered by significant bias and demonstrate association without an estimation of causation.^39^

## Challenges of Target Trial Emulation

There are several disadvantages of using target trial emulation that may hinder widespread acceptability and use in IBD research. The lack of randomization means these study designs remain subject to bias from unmeasured confounding factors, even if known confounders, such as prior advanced therapy exposure or smoking status in Crohn’s disease, are accounted for. Potential confounders should be identified a priori and statistically adjusted for to attempt to mimic the process of randomization.^11^ Patients with IBD outside the context of trials are often treated with drug regimens that can vary considerably over time, such as dose escalation/de-escalation or the addition of immunomodulating or corticosteroid therapy, which need to be considered to avoid bias.^40^ Adjusting for such time-varying confounders is possible but requires more complex statistical approaches, such as parametric g-formula and inverse probability of treatment-weighting methods. The lack of blinding and knowledge of treatment assignment is another pitfall because for observational data, investigators will be aware of which treatment has been assigned. Therefore, target trial emulations should not be placebo-controlled and need to be pragmatically designed.

The quality of data obtained from observational studies and healthcare records should be considered a potential limitation. These data are often subject to missingness, misclassification from incorrect coding, and general inaccuracies, unlike data from clinical trials, which are more thoroughly collected. A common reason for studies that fail to mimic the target trial is due to missing or poor-quality data, particularly if this affects data for potential confounders. This highlights the need to establish and maintain well-designed databases for IBD patients to accurately record demographics, phenotypic data and postintervention outcome measures to facilitate collaborative observational research.

Studies need to have a clear protocol, follow it, and ensure this is published alongside study findings to enable critique, but unfortunately, this happens less commonly than one would think. In a systematic review of 200 studies that report emulating a randomized trial, 57% did not describe the target trial protocol, and 43% did not report the key features of how the target trial was emulated.^7^ An important hurdle of considering this study design is that it is novel, and hence researchers may lack the practical knowledge and experience of how best to design and deliver it.

Another challenge is that only a small proportion of patients identified in observational records are likely to be eligible for inclusion in a study that mimics a target trial.^41^ For example, in a recent comparative effectiveness real-world study of adjuvant chemotherapy in advanced colon cancer, only 16% of patients were eligible for inclusion compared with the actual target trial.^41^ This can reduce the power to detect a difference between the effect estimates between the 2 studies. It is important to consider this beforehand to maximize data availability by, for example, recruiting patients from multiple rather than single centers, or selecting a therapy that has been used in routine clinical practice for an extended period of time.

Finally, it may not be necessary, or indeed possible, to generate results comparable to RCTs. Real-world evidence has significant merit on its own given its strong external validity. It is more reflective of routine clinical care and complements RCT data, which are obtained from restrictive populations. If target trials are emulated, there may be other clinical or methodological explanations for the observations seen, even if each trial component is emulated as close as possible.^42,43^ This includes the limitations of comparing differing study designs, outcome definitions, follow-up periods, and analytical choices made. There are also limitations of comparing trial subjects and research investigators that may not be representative of everyday clinical practice. For example, the variability of treatment adherence from subjects, or the variability in how the end point is recorded (driven by experts in trials, eg, centrally read endoscopy), is difficult to replicate.

## Conclusions

Conducting a randomized trial remains the optimum way to test the safety and effectiveness of an intervention, but this is not always possible. Accurate estimation of treatment effects from observational data is difficult due to bias. Target trial emulation is a causal inference framework that emulates RCTs using nonrandomized observational data. This methodological strategy, even if used partially with some of the concepts emulated (eg, time zero) could improve the quality of IBD observational studies by clarifying design choices and reducing bias. This approach has been shown to produce effect estimates comparable to RCTs across several medical disciplines and may be used to test the effect of IBD interventions where RCTs are impractical. Target trial emulation is especially relevant in the field of IBD as it permits the study of IBD subpopulations that have been and continue to be underrepresented in pivotal trials. One of the biggest drawbacks includes residual confounding due to the lack of randomization and the requirement for complex statistical approaches to ensure overall bias is kept to a minimum. Nonetheless, such study designs should be used to improve the quality of observational research and prevent poor conclusions drawn regarding causality. Reporting guidelines are currently in development to help standardize this process.^44^

## Data Availability

Data sharing not applicable—no new data generated.

## Abbreviations:

CD: Crohn’s disease
IBD: inflammatory bowel disease
IL: interleukin
RCT: randomized controlled trials
UC: ulcerative colitis
